# An unusual gall-bladder polyp - site of metastatic renal cell carcinoma: a case report

**DOI:** 10.1186/1757-1626-2-172

**Published:** 2009-10-29

**Authors:** Sandeep Patel, Bassel Zebian, Shashank Gurjar, Nevil Pavithran, Krishna Singh, Tom Liston, Jerry Grant

**Affiliations:** 1 Medway Maritime Hospital, Kent, UK; 2 King's College Hospital, London, UK; 3 Worthing Hospital, Worthing, UK

## Abstract

We report the case of a 64 year old woman who presented with symptomatology of gallstone disease but was radiologically shown to have a polyp within the gallbladder. Upon resection this was shown to be a metastasis from renal cell carcinoma for which she had had a nephrectomy six years previously.

## Case presentation

A 64 year old Caucasian female presented with recurrent right upper quadrant pain that radiated through to her back, often associated with ingestion of fatty foods. She also complained of unrelated episodes of regurgitation and vomiting. Of note, she had previously been diagnosed with a renal cell carcinoma and undergone a left nephrectomy, approximately six years ago. At the time, a follow-up ultrasound scan of her abdomen had revealed incidental gallstones. Abdominal examination revealed a soft abdomen with no localised tenderness or palpable masses. A working diagnosis of cholecystitis was made.

A repeat ultrasound scan confirmed the presence of gallstones, but also noted a 3 cm polyp within the gall-bladder. This appeared to be benign but a CT scan was carried out for further evaluation. This showed an irregular polyp centred within the gall-bladder with a tiny accompanying nodule on its medial surface.

**Figure 1:**
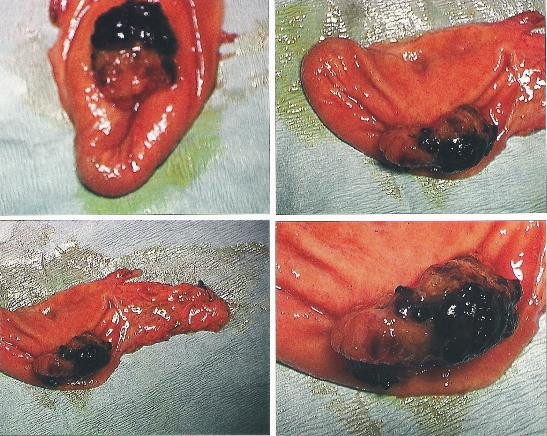
**Intraoperative photograph showing gallbladder dissection and no infiltration into liver**.

**Figure 2:**
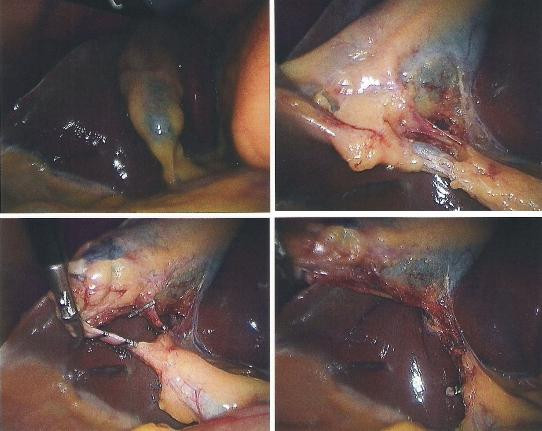
**Macroscopic image of resected specimen with polyp visible**.

The patient underwent a laparoscopic cholecystectomy without complication. At surgery, there was no evidence of metastatic disease. The gall-bladder was found to contain numerous small calculi and a polyp as demonstrated by previous imaging. The polyp was entirely within the gall-bladder and not extending into the liver (Figure 1, 2).

Histological examination revealed that the polyp was in fact a clear cell carcinoma consistent with metastatic renal cell carcinoma, which was morphologically similar to the renal tumour excised in 2001. She made an uneventful recovery and was discharged with planned follow-up by way of interval surveillance CT imaging.

## Discussion

Metastases to the gall-bladder are very rare and and in most cases, the primary tumour is a malignant melanoma [1,2]. Renal cell carcinomas account for approximately 3% of all adult malignancies and have the potential to metastasise to almost any site in the body [3]. The commonest sites are lung, soft tissue, bone, liver, skin and the central nervous system [4,5]. Metastases to the gall-bladder are extremely uncommon and usually detected only at autopsy. Clinically, patients present with symptoms and signs typically associated with gallstone disease. This can occur many years after the initial nephrectomy. The nature of polypoidal masses within a gall-bladder can be difficult to elucidate [6]. Limani et al [2] stated that preoperative imaging was futile in differentiating between primary and secondary tumours of the gall-bladder. They reported that primary tumours often coexisted with gallstones whilst metastatic disease was associated with an acalculus gall-bladder. The latter are often associated with hypervascularity on diagnostic imaging [7].

Metastatic gall-bladder involvement is associated with a poorer prognosis. 23 cases of metastatic renal cell carcinoma of the gall-bladder have been reported - of these 9 remained free of recurrent disease post cholecystectomy, with the longest follow up interval being 6 years [8]. Although metastases are a bad prognostic factor, it seems that patients with solitary metastases such as in the gallbladder have an improvement in survival after resection of the lesion [4], usually by way of a laparoscopic cholecystectomy.

## Abbreviations

CT: Computed tomography.

## Consent

Written informed consent was obtained from the patient for publication of this case report and accompanying images. A copy of the written consent is available for review by the Editor-in-Chief of this journal.

## Competing interests

The authors declare that they have no competing interests.

## Authors' contributions

BZ and NP wrote the paper, SP and SG edited and revised the paper, KS and TL performed the surgery, JG interpreted the specimen. All authors have read and approved the final manuscript.
